# Enzymatic Protein Immobilization for Nanobody Array

**DOI:** 10.3390/molecules29020366

**Published:** 2024-01-11

**Authors:** Zhuojian Lu, Rui Ge, Bin Zheng, Peng Zheng

**Affiliations:** State Key Laboratory of Coordination Chemistry, Chemistry and Biomedicine Innovation Center (ChemBIC), School of Chemistry and Chemical Engineering, Nanjing University, Nanjing 210023, China; dg20240070@smail.nju.edu.cn (Z.L.); mf21240005@smail.nju.edu.cn (R.G.); mg21240135@smail.nju.edu.cn (B.Z.)

**Keywords:** protein immobilization, enzymatic ligation, *Oa*AEP1, nanobody

## Abstract

Antibody arrays play a pivotal role in the detection and quantification of biomolecules, with their effectiveness largely dependent on efficient protein immobilization. Traditional methods often use heterobifunctional cross-linking reagents for attaching functional residues in proteins to corresponding chemical groups on the substrate surface. However, this method does not control the antibody’s anchoring point and orientation, potentially leading to reduced binding efficiency and overall performance. Another method using anti-antibodies as intermediate molecules to control the orientation can be used but it demonstrates lower efficiency. Here, we demonstrate a site-specific protein immobilization strategy utilizing *Oa*AEP1 (asparaginyl endopeptidase) for building a nanobody array. Moreover, we used a nanobody-targeting enhanced green fluorescent protein (eGFP) as the model system to validate the protein immobilization method for building a nanobody array. Finally, by rapidly enriching eGFP, this method further highlights its potential for rapid diagnostic applications. This approach, characterized by its simplicity, high efficiency, and specificity, offers an advancement in the development of surface-modified protein arrays. It promises to enhance the sensitivity and accuracy of biomolecule detection, paving the way for broader applications in various research and diagnostic fields.

## 1. Introduction

Antibody arrays have emerged as powerful tools in biomolecule detection and quantification and are widely applicable in basic research and clinical diagnostics [1,2,3,4]. Its effectiveness lies in the antibody’s capability to precisely detect target molecule [5,6]. Successful antibody arrays depend on the efficient immobilization of antibodies while preserving their binding specificity and sensitivity [7,8]. Classic immobilization strategies typically use a heterobifunctional cross-linking reagent capable of reacting with cysteines or lysines in the protein on one side and reacting with amino or carboxyl groups on the surface on the other site [9,10]. However, a reciprocal decrease in site specificity occurs for amine- and carboxyl-specific crosslinkers as the number of corresponding functional groups increases, resulting in orientation loss [11,12]. Conversely, linking via sulfhydryl or aldehyde groups on antibodies is more selective and offers improved orientation. However, creating sulfhydryl and aldehyde groups on antibodies involves relatively aggressive conditions, potentially triggering undesired reactions and impacting the binding of antibodies to their target antigens [13,14]. An alternative approach employs anti-antibodies for immobilization [15,16]. It ensures that the binding regions of antibodies remain distant from the substrate surface, thus avoiding most of the adverse effects on the binding efficiency of the antibody [17,18]. This will negatively affect the lifetime of the antibody arrays and result in an increased distance between the capture component and the antibody array surface [19]. In addition, this method is easily influenced by pH and ionic strength, leading to the occurrence of multiple orientations [20]. Furthermore, the adsorption process involves dynamic interactions between the solution and the bound phases of antibodies. Besides random orientation, immobilized antibodies can readily leach out in the presence of another protein with a higher charge or more hydrophobic pockets [21]. This is due to relatively weak and reversible interactions, leading to inefficient antigen binding even when a higher quantity of antibodies is adsorbed onto the surfaces [22].

To address the challenges associated with immobilized antibodies, we propose an enzymatic method for the site-specific and covalent immobilization of nanobodies on surfaces. Enzymatic ligation, based on site-specific coupling, effectively addresses the issue of non-specific protein modification [23,24,25]. For example, Sortase A and *Oa*AEP1 can recognize two specific peptide sequences and ligate them together [26,27,28]. By introducing the specific peptide sequence at one end of the target protein through genetic engineering, additional functional group modification processes are not required, enabling specific protein conjugation [29]. For instance, Sortase A (SrtA) recognizes the *C*-terminal LPXTG sequence and ligates it to the (G)n *N*-terminal of another protein [30,31,32]. This reaction has been applied in protein conjugation and surface modification processes [33,34]. Similarly, *Oa*AEP1, an asparaginyl endopeptidase, recognizes NGL at the *C*-terminus and ligates it to a GL- *N*-terminus [35,36,37]. Its C247A mutant achieves a high catalytic efficiency of approximately 70% with reduced hydrolysis, further underscoring the potential of this enzymatic approach [38]. The shorter recognition sequence NGL not only allows for precise and targeted enzymatic ligation, thereby minimizing off-target effects, but also reduces the impact of introducing additional amino acids on protein expression and activity [39,40,41]. With its specific recognition, versatility, enhanced catalytic efficiency, and reduced hydrolysis, this method has found applications in protein conjugation and single-molecule force spectroscopy [42,43,44,45,46]. Therefore, we selected *Oa*AEP1 for enzymatically immobilizing proteins to construct a nanobody array.

**Figure 1:**
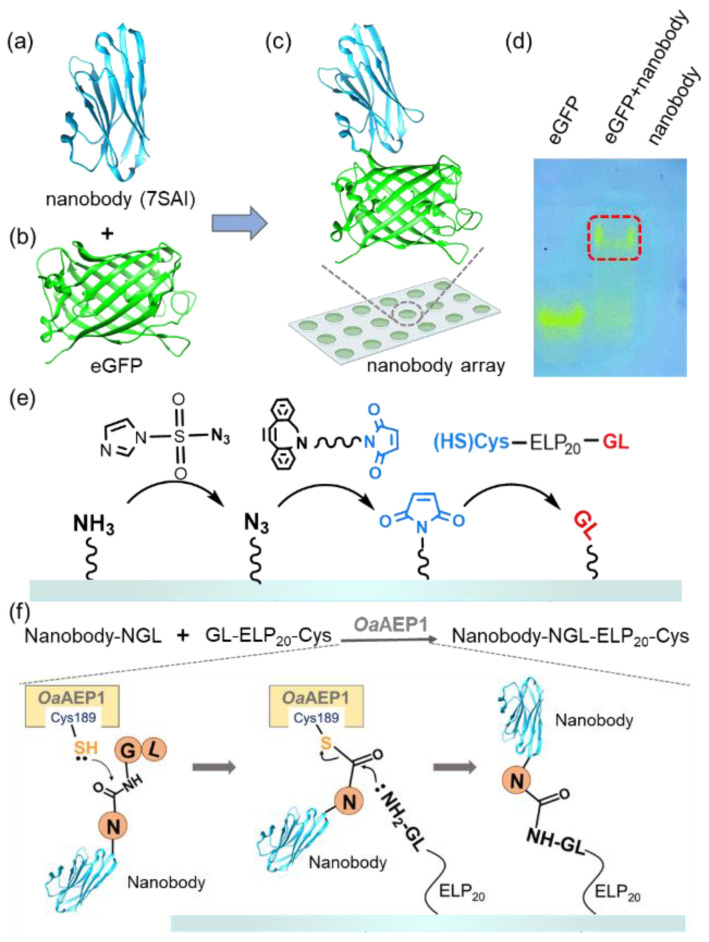
(**a**–**c**) The structure of the nanobody (PDB 7SAI) targeting eGFP is shown. (**d**) Native PAGE gel confirmed the interaction between the nanobody and eGFP. The migration of visible eGFP bands indicated the complex formation, which is highlighted by the red box. (**e**) Schematic representation shows the addition of a GL-peptide on amino-functionalized glass. First, the surface was coated with N_3_ from NH_2_; then, the Maleimide group was added. Finally, the target peptide GL was added [45]. (**f**) Nanobody with an NGL tag was immobilized on the GL-functionalized surface catalyzed by *Oa*AEP1 [27].

To illustrate this strategy, we used nanobody, single-domain antibodies from camelid HcAbs known for their small size and high expression [47,48]. Their smaller molecular weight and higher solubility enable dense surface immobilization on surfaces at higher concentrations without the aggregation propensity [49,50,51]. It enhances the quantity of antibody immobilization on the surface and increases the sensitivity of antibody arrays [52,53]. Despite their size, nanobodies maintain a strong antigen-binding affinity [54]. For instance, the nanobody (7SAI) binds toeGFP (Figure 1a,b and Figure S1a,b) with a nanomolar-level affinity (Figure 1c) [55]. This binding affinity was further validated through native PAGE (Figure 1d) and we used this nanobody for demonstration in this work.

## 2. Results and Discussion

The process of our nanobody immobilization method is illustrated in Figure 1e,f. First, a maleimide-modified glass substrate was prepared using a click chemistry approach as previously reported. Subsequently, GL-ELP_20_-Cys is immobilized on the glass through the interaction between maleimide and thiol, enabling the GL-functionalized substrate to participate in the subsequent enzymatic ligation catalyzed by *Oa*AEP1 [56] (Figure 1e and Figure S2). Following the recognition of the *C*-terminal-NGL sequence of the nanobody, the thiol group of C189 of *Oa*AEP1 nucleophilically attacks the carbonyl group between Asn and Gly, forming a thioester intermediate (Figure 1f) [57]. Subsequently, the amine group of NH_2_-GL-ELP_20_ on the substrate attacks the thioester intermediate, resulting in a peptide bond and efficient nanobody immobilization. Moreover, using GL-ELP_20_-Cys peptide segments, approximately 20 nm in length, provides space and flexibility for the nanobody to prevent a reduction in binding affinity due to steric hindrance. Simultaneously, this method also minimizes non-specific adsorption, enhancing subsequent detection steps’ sensitivity.

**Figure 2:**
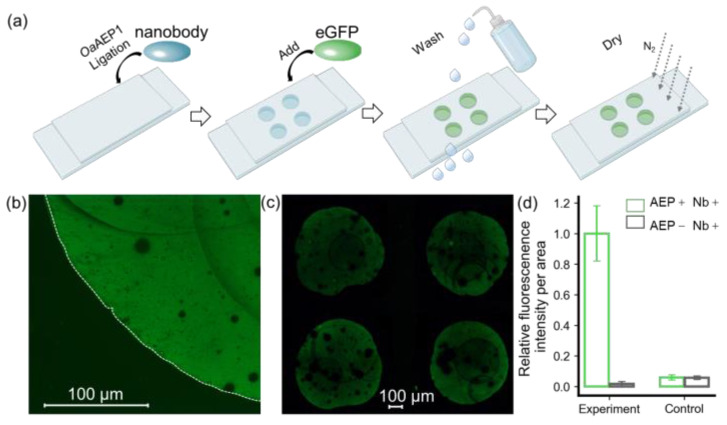
Verification of immobilized eGFP by fluorescent imaging. (**a**) A step-by step procedure for constructing an antibody array to recruit eGFP. (**b**) Hand-pipetted immobilized eGFP on glass exhibits a distinct fluorescent signal, with its boundary outlined by a dashed line in the bright-field image. (**c**) eGFP was added in a 2 × 2 nanobody microarray, resulting in a clear fluorescence pattern. (**d**) Quantification of fluorescent intensity per unit area for these methods, which includes an experiment and a control, was used to confirm the feasibility of this approach. ImageJ was used for quantification, and the values were normalized. The histograms depict the relative intensity of eGFP in the presence (green) and absence (black) of *Oa*AEP1.

Here, we used a nanobody targeting eGFP as the model system to validate the protein immobilization method for building a nanobody array (Figure S1). As shown in Figure 2, a nanobody-NGL and *Oa*AEP1 mixture was pipetted onto the GL-functionalized substrate to create a 2 × 2 nanobody array. Subsequent washing with a high-salt buffer and deionized water was performed to remove the unreacted nanobody, *Oa*AEP1, and non-covalently adsorbed contaminants. The eGFP solution was pipetted onto the areas with the immobilized nanobody and immediately washed off to remove unbound eGFP (Figure 2a). Using a fluorescence microscope with an excitation wavelength of 490 nm, the fluorescence pattern on the glass substrate was observed, revealing a distinct boundary generated by eGFP firmly recruited by the nanobody (Figure 2b). A 2 × 2 fluorescence array could be observed in a wide field of view, indicating the successful preparation of the nanobody array (Figure 2c). Quantitative analysis and normalization of fluorescence intensity, as assessed with ImageJ, are shown in Figure 2d. The ratio of fluorescence intensity per unit area within the fluorescence pattern to the external background was 98% versus 2% (*p* = 0.000026 < 0.01), highlighting the method’s high sensitivity (Figure 2d).

Furthermore, we conducted a comparative analysis using a non-functionalized glass substrate, which retains hydroxyl groups on the surface. Remarkably, the fluorescence intensity on this substrate was observed to be 7%, in contrast to a mere 2% (*p* = 0.00003056 < 0.01) on the GL-functionalized substrate (Figure S3). This reduction in background signal notably enhances the method’s resistance to interference. These results affirm that the enzymatic ligation reaction catalyzed by *Oa*AEP1 not only achieves nanobody arrays with lower background signals but also enhances specificity.

**Figure 3:**
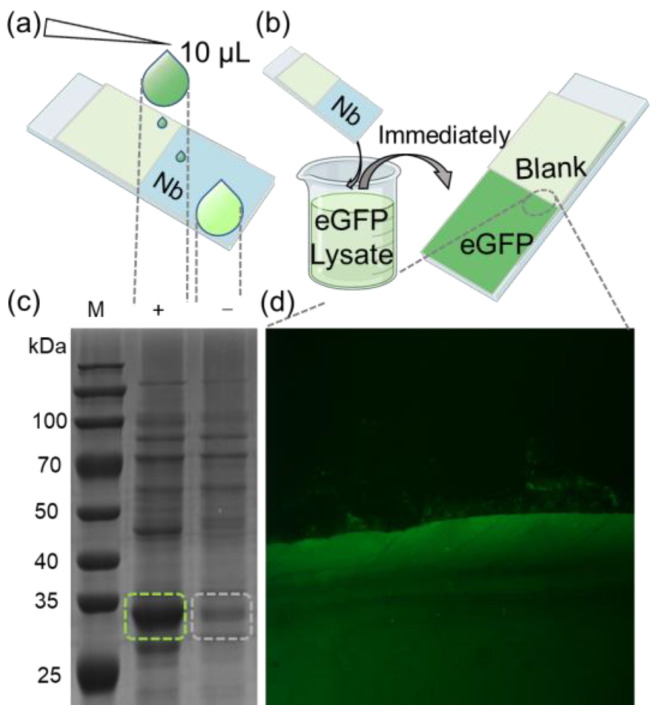
(**a**) Schematic diagram of trace detection of enriched eGFP protein. (**b**) Diagram of rapid recruitment of eGFP protein from lysate. (**c**) Cell lysates derived from *E. coli* cells transformed with an eGFP-expressing plasmid were subjected to SDS-PAGE analysis, with “(+)” representing the original cell lysate and “(−)” representing the lysate from nanobody array treatment. The green and gray dashed boxes represent the bands of the eGFP protein before and after the reaction of the lysate with the nanobody array. (**d**) eGFP was spotted onto the immobilized nanobody. Recruitment boundary was clearly observed between eGFP and blank (magnified section indicated by dashed lines).

To further demonstrate the efficacy of our method, we applied it to enrich target proteins from cell lysates. A glass slip was partially coated with the eGFP nanobody, and 10 μL of bacterial lysate from eGFP-overexpressing in *E. coli* was applied onto its surface. Subsequently, the residual liquid on the substrate was collected for SDS-PAGE analysis and validation (Figure 3a). The results indicated that the bands corresponding to eGFP in the collected liquid were significantly fainter compared to those in the original lysate. For semi-quantitative analysis, grayscale values of the SDS-PAGE lanes were assessed from top to bottom using ImageJ (Figure S4a). The findings revealed that the grayscale values of eGFP in the collected liquid were markedly lower compared to the cell lysate (Figure S4b).

Next, the glass slip was fully immersed in a bacterial lysate containing eGFP, then quickly retrieved (Figure 3b). After drying the substrate, fluorescence imaging was performed following the previously mentioned procedure. A substantial fluorescence signal was observed in the nanobody-immobilized area, with a distinct boundary between the nanobody-bound and the blank areas (Figure 3b). Further visualization clearly illustrates the high efficiency of nanobody in enriching eGFP. The combined results from the SDS-PAGE and fluorescence imaging corroborate that the nanobody-immobilized substrate can efficiently enrich target protein from complex mixtures, demonstrating high sensitivity. This method streamlines the experimental process, offering a more compatible and convenient process for various applications, including antibody array preparation for ELISA and immunoprecipitation.

## 3. Materials and Methods

### 3.1. Materials

All reagents utilized in the experiment were procured from reputable commercial suppliers. (3-Aminopropyl) triethoxysilane (APTES, Sigma Aldrich, Darmstadt, Germany), Imidazole-1-sulfonyl Azide Hydrochloride (ImSO_2_N_3_∙HCl, Abydos Scientific, Shanghai, China) and DBCO (dibenzocyclooctyne)-PEG_4_-maleimide (Biocone, Shanghai, China) were stored and handled within a light-protected environment to ensure their stability. K_2_CO_3_ and CuSO_4_ were obtained from Aladdin (Shanghai, China). Chemical solvents, such as toluene, ethanol, and dimethyl sulfoxide (DMSO), were acquired from Sinopharm Chemical Reagent Co. Ltd, Beijing, China. Aqueous solutions were prepared using Milli-Q water (18.2 MΩ/cm, 0.22-µm filter) to ensure high purity. Luria-Bertani (LB) medium and agar plates (Sangon Biotech, Shanghai, China) were employed for the cultivation of *E. coli.* The optical density of bacterial cultures at 600 nm (OD_600_) was determined using a Metash UV-5500PC (Metash, Shanghai, China) spectrophotometer. Fluorescent imaging of eGFP was conducted with a fluorescent microscope (Olympus IX73, OCPNY, Tokyo, Japan). Glass surfaces were activated with a plasma cleaner. Protein concentrations were determined using a Nanodrop 2000 spectrophotometer (Thermo Fisher, Waltham, MA, USA), ensuring precise measurements for downstream experimental procedures.

### 3.2. Protein Engineering

#### 3.2.1. Expression and Purification of eGFP and Its Corresponding Nanobody

The genetic sequences corresponding to the nanobody (7SAI) and eGFP were procured from Genscript (Piscataway, NJ, USA) and subsequently inserted into pET21b and pET22b vectors, respectively. The resultant constructs, namely pET21b-eGFP and pET22b-nanobody (7SAI)-NGL, were introduced into BL21(DE3) cells for expression. Cultivation was carried out using LB media supplemented with 100 µg/mL ampicillin sodium salt, with the bacterial cultures maintained in a shaking incubator at 37 °C. Upon reaching an optical density at OD_600_ of 0.6–0.8, induction of protein expression was initiated by the addition of isopropyl β-d-thiogalactoside (IPTG) to the culture medium at a final concentration of 0.4 mM. Subsequent to induction, eGFP was cultivated for an additional 4 h at 37 °C, while the nanobody culture was prolonged overnight at 18 °C. Following expression, bacterial cultures were subjected to centrifugation (8000 rpm, 10 min, 4 °C) and subsequently stored at −80 °C for the subsequent purification processes.

Cell pellets were resuspended in lysis buffer (50 mM Tris, pH 7.4, 1 mM PMSF) and lysed using a homogenizer (UH-03, Union-biotech, Shanghai, China) with 4 °C water circulation. Subsequently, the cell lysate was centrifuged (18,000 rpm, 15 min, 4 °C), and the supernatant was incubated with Co-NTA affinity beads (TALON, Zhongshan, China) pre-equilibrated with a washing buffer (20 mM Tris, 400 mM NaCl, 2 mM imidazole, pH 7.4). The bound protein was washed with the washing buffer and then eluted with an elution buffer (20 mM Tris, 400 mM NaCl, 250 mM imidazole, pH 7.4). The buffer of eluted proteins was exchanged to an experimental buffer (100 mM Tris, 100 mM NaCl, pH 7.4) using ultrafiltration. The eluted nanobody was subsequently dialyzed in the dialysis buffer (e.g., 1× DPBS, pH 7.4). A HiLoad 16/600 Superdex 200 pg column (GE Healthcare, Little Chalfont, UK) on an AKTA HPLC protein purification system (GE Healthcare, Little Chalfont, UK) was used to further purify the nanobody. The purified nanobody and eGFP were analyzed by SDS-PAGE (Figure S1a). Following this, no distinct impurity bands were observed through SDS-PAGE. Subsequently, the purified nanobody and eGFP underwent additional purification. Finally, they were filtered using a 0.2-micrometer filter (Millex, Darmstadt, Germany) for sterilization and stored at −80 °C for use.

#### 3.2.2. Expression, Purification, and Self-Activation of *Oa*AEP1

This study involved the strategic insertion of a gene into the NcoI-NdeI coding region of the pET-28b(+) vector, followed by amplification. The recombinant expression of *Oa*AEP1 in *E. coli*, specifically in the BL21(DE3) strain, adhered to previously established protocols. The initial seed culture was incubated at a controlled temperature of 37 °C for a duration of 16–18 h. Subsequently, this culture was transferred to 800 mL of LB medium resistant to kanamycin and maintained at 37 °C for approximately 3 h. A subsequent adjustment in temperature to 16 °C was implemented, accompanied by the induction of protein expression using 0.4 mM IPTG for a period of 20 h. Post-expression, the cells were collected through centrifugation at 8000 rpm for 10 min at a temperature of 4 °C. The harvested cells were then resuspended in a lysis buffer composed of 50 mM Tris-HCl (pH 7.4), 150 mM NaCl, 0.05% (*v*/*v*) CHAPS, and 10% (*v*/*v*) glycerol. Cell lysis was achieved through homogenization over a duration of 3 min under a pressure of 750 bar. The resultant supernatant was further subjected to purification via metal affinity chromatography using a Ni-NTA column (TALON). Proteins containing His tags, which adhered to the column, were subsequently eluted using a linear gradient of imidazole ranging from 0 to 250 mM, in a buffer containing 50 mM Tris-HCl (pH 8.0), 150 mM NaCl, 0.05% (*v*/*v*) CHAPS, and 10% (*v*/*v*) glycerol. The elution fractions from the Ni-NTA column containing the target protein were then diluted with buffer A (0 mM NaCl) at a ratio of 1:5 and introduced into two 5 mL HiTrap Q Sepharose high-performance columns connected in series (GE Healthcare; 2 mL sample per ml of resin). The bound proteins were eluted using a continuous salt gradient ranging from 0 to 30% of buffer B, composed of 20 mM Bis-Tris propane and 2 M NaCl at pH 7.4 The final purification step involved passing the protein through an SEC column pre-equilibrated with PBS buffer.

The process of self-activating *Oa*AEP1 involved the addition of 1 mM EDTA and 0.5 mM Tris (2-carboxyethyl) phosphine hydrochloride to the immature protein. This step was followed by an adjustment of the solution’s pH to 4, achieved through the addition of glacial acetic acid. These pooled fractions were then subjected to an incubation period of 16 h at either room temperature or at a controlled temperature of 37 °C. The adjustment to this specific pH facilitated protein precipitation, effectively enabling the separation and removal of the majority of contaminating proteins via centrifugation. The proteins that were successfully activated through this procedure were subsequently concentrated through ultracentrifugation, utilizing a concentrator with a molecular weight cutoff of 30 kDa (Sartorius brand, Göttingen, Germany). Subsequently, these proteins were utilized to catalyze POI-NGL and GL-POI, leading to the identification of distinctive ligation protein bands on SDS-PAGE. Following this, they were stored at −80 °C for future use.

### 3.3. Surface Preparation

Surface modification of 24 mm × 76 mm glass slides (Sail Brand, Yancheng, China) was performed through a series of steps [45]. The slides underwent plasma cleaning and activation for 20 min. Then, they were immersed in a 10% (*v*/*v*) solution of APTES in toluene and maintained in the dark at room temperature (~25 °C) for one hour. To remove excess APTES, the slides were washed with anhydrous ethanol (99.5% purity) and dried using a stream of nitrogen gas (N_2_). The slides were then heated in an oven at 80 °C for 15 min, followed by cooling to room temperature (Figure S2a). At this point, the surface of the glass slides was successfully modified with amino groups.

Next, approximately 50 µL of an aqueous solution containing 2 mM ImSO_2_N_3_, 4 mM K_2_CO_3_, and 20 µM CuSO_4_ was added to the aminosilane-functionalized glass cover slips. This mixture was incubated at room temperature in the dark for one hour (Figure S2a). The slides were then thoroughly washed with deionized water, followed by 99.5% ethanol and drying under N_2_ gas, resulting in an azide-functionalized surface (Figure S2b).

Approximately 50 μL of a DBCO-PEG_4_-maleimide solution (2 mM, dissolved in DMSO) was added to the azide-functionalized glass cover slips. They were then incubated at 37 °C in the dark for one hour (Figure S2c). The DBCO reacts with the azide groups to introduce maleimide groups for subsequent modifications. After this reaction, the cover slips were rinsed with 99.5% ethanol to remove unbound DBCO-PEG_4_-maleimide, and dried under N_2_ gas.

And then, approximately 50 μL of GL-ELP_20_-C solution (180 μM) was added to the maleimide-functionalized glass cover slips, which were left at room temperature for about 3 h (Figure S2d) [56]. The slides were then washed with 50 mL of high-salt buffer solution (100 mM Tris, 1 M NaCl, pH 7.4) and rinsed with deionized water to remove residual buffer, followed by drying under a nitrogen gas flow. The GL-ELP_20_-coated slides were ready for immediate use or could be stored at −20 °C for up to six weeks (Figure S2e).

### 3.4. Protein Immobilization

To immobilize the nanobodies, we added a mixture of 10 µL containing 30 µM nanobody and 1 µM *Oa*AEP1 solution onto glass slides modified with GL-ELP_20_- (see Figure S2f) [42]. Subsequently, a 2 × 2 protein microarray was fabricated using the same procedure. The entire reaction process was incubated at room temperature for 30 min. Afterwards, the glass slides underwent an extensive washing process, including washing with 50 mL of high-salt buffer (containing 100 mM Tris, 1 M NaCl, pH 7.4) and rinsing with 50 mL of deionized water, performed three times to remove non-specifically bound proteins. Finally, the slides were then gently dried using nitrogen gas.

In the control setup, during the final step of nanobody immobilization, *Oa*AEP1 was excluded, and the substrate was not coated with GL-ELP_20_. Other conditions, including eGFP concentration and incubation time, remained consistent with the experimental group. Subsequently, fluorescence imaging was performed on the substrates prepared under these conditions.

### 3.5. Fluorescence Imaging

Imaging was performed on a microarray enriched with eGFP using an Olympus IX73 microscope. The microscope was equipped with a ×10 magnification objective lens and a numerical aperture (NA) of 0.3. The imaging process included using a 488 nm excitation wavelength, observing fluorescence after filtering through a 500 to 550 nm filter, exposing each image for 15 s, and then moving the platform to capture multiple images, thereby composing a complete fluorescence matrix image. The stitching and analysis of the fluorescence images were conducted using ImageJ 1.54f software.

### 3.6. Data Analysis

#### 3.6.1. Analysis of Fluorescence Density

In the study, ImageJ software was employed for the quantitative analysis of fluorescence images. The luminescent regions in the images are circular areas where eGFP is captured by nanobodies immobilized on the substrate surface. These regions are distinctly illuminated against a non-luminous background.

The analytical approach commenced with the selection of specific areas within the fluorescence images. This was achieved by employing the rectangular selection tool in ImageJ, enabling precise delineation of both illuminated and non-illuminated regions. The selection process focused on capturing a representative sample of the luminescent circles, as well as adjacent non-luminous areas, to facilitate a comparative grayscale analysis.

Following the selection, the grayscale values of these defined areas were quantitatively analyzed. This involved the assessment of grayscale intensity within each selected rectangle, providing a measure of the relative fluorescence intensity in the luminescent regions compared to the background. The analysis was meticulously executed for various regions across the sample, including *t*-tests on the fluorescence density of illuminated and non-illuminated regions at different locations. This ensured a comprehensive understanding of the spatial distribution of eGFP fluorescence.

This methodical approach allowed for a detailed quantitative evaluation of the fluorescence intensity, contributing significantly to the understanding of the binding efficiency and spatial distribution of eGFP on the antibody-modified substrate. The technique’s precision and reproducibility make it a valuable tool for similar studies in fluorescence microscopy and protein interaction analysis.

#### 3.6.2. SDS-PAGE Grayscale Analysis

Stripe grayscale values using ImageJ software. The initial phase involves acquiring the target image via ImageJ’s interface, either through the ‘File’ menu or by direct drag-and-drop, which triggers automatic image recognition. In scenarios where the image features a black background with white stripes, an initial step of color inversion may be necessary, accessible under the ‘Edit’ menu. Following this, the image undergoes conversion to an 8-bit grayscale format, a critical transformation that enables the identification of 256 unique grayscale shades, ranging from 0 (black) to 255 (white), essential for nuanced analysis.

A key aspect of this methodology is the elimination of background interference, achieved by using the ‘Process’ > ‘Subtract Background’ function in ImageJ, with settings adjusted to a rolling ball radius of 50.0 pixels and a preference for light background subtraction. The analytical process then involves the precise manual selection of individual stripes using ImageJ’s rectangular selection tool. Upon selection, the command buttons are used to generate a profile plot that graphically represents the grayscale intensity distribution across the chosen area. This plot requires manual closure to maintain an interactive workflow. The procedure is meticulously repeated for each stripe of interest, ensuring a comprehensive and methodical analysis of grayscale variations.

## 4. Conclusions

In this study, we achieved site-specific immobilization of nanobody-NAL onto GL-functionalized substrates with high efficiency. This achievement aligns with our broader exploration, where we introduce an innovative enzymatic approach for the site-specific and covalent immobilization of nanobodies on various surfaces [45]. This approach effectively addresses the challenges associated with traditional methods for antibody arrays. By introducing a specific peptide sequence at one end of the target protein through genetic engineering, this method enables specific protein conjugation without the need for additional functional group modification processes. The relatively short recognition sequences of NGL and GL, integral to *Oa*AEP1, facilitated this process without compromising the intrinsic binding capabilities of the nanobody. This was validated by constructing an eGFP fluorescence array (Figure 2). Moreover, this method facilitates the rapid enrichment of target proteins from complex biological systems. Further elucidation through enzymatic protein immobilization for nanobody arrays underscores its practical utility (Figure 3).

A significant challenge in assembling a nanobody matrix is the need to present each different protein in a conformation and orientation that ensures biological activity [58]. For antibodies, this challenge means maximizing the proportion of immobilized proteins that bind to the target antigen. Ideally, all immobilized antibodies would bind to the antigen with the same affinity. In practice with previous methods, less than half of the immobilized antibodies were functional, and even then, they bound to the antigen with a range of affinities [59]. We anticipate that this fraction of activity should be minimized when antibodies are uniformly oriented and immobilized to maximize the number of bound antigen molecules, without introducing interactions between adjacent antibody–antigen complexes.

This paper introduces a method that meets these objectives and provides an effective strategy for preparing a nanobody matrix. The *Oa*AEP1 enzyme-linked catalysis strategy ensures the uniform orientation of immobilized antibodies. Firstly, nanobodies are constructed through genetic engineering with specific recognition sequences, allowing them to react with the substrate surface more flexibly and directionally, ensuring that the nanobodies are fully oriented in the same direction. This allows for the adoption of the same favorable conformation to optimize binding with the antigen. Secondly, the enzyme-linked reaction fully realizes the site-specific immobilization of nanobodies, ensuring the maximization of immobilization efficiency. This approach somewhat eliminates the interference of non-specific proteins, improving the performance of the entire nanobody matrix. Additionally, this method allows for direct control of the density of immobilized proteins. Since the immobilization of nanobodies requires a covalent reaction with the ELP on the substrate surface, this density can be easily controlled. Another key consideration in antibody immobilization is spatial hindrance [60]. Traditional antibodies, due to their larger size, often face challenges in increasing modification density. This can lead to interactions between antibody molecules, resulting in signal interference and reduced detection sensitivity. In contrast, nanobody arrays, leveraging the unique attributes of nanobodies, have immense potential in revolutionizing disease marker detection. These arrays are distinguished by their compact size and superior affinity, highlighting their ability to transform the field in multiple ways. Firstly, the augmented sensitivity and specificity of nanobody arrays facilitate precise recognition of disease-specific biomarkers, significantly reducing the risk of erroneous results. This feature has far-reaching implications, ranging from accurate disease identification to potential prognostic insights. Moreover, the increased sensitivity of nanobody arrays paves the way for early disease diagnosis, a crucial element in enhancing patient outcomes and influencing the course of various health conditions. This is particularly vital in scenarios where early intervention is key. Additionally, the multiplexing capabilities of nanobody arrays amplify their transformative impact. By allowing the simultaneous detection of multiple biomarkers in a single assay, these arrays provide a comprehensive view of diseases, especially when a spectrum of biomarkers is essential for the diagnostic process.

In addition to their role in diagnostics, nanobody arrays are poised to revolutionize point-of-care applications, offering rapid and on-site diagnostic capabilities. This advancement is particularly significant in resource-constrained settings and situations where immediate results are crucial for effective patient management. A notable demonstration of these advantages is the successful enrichment of the target protein eGFP in cell lysates. As previously mentioned, this achievement underscores the ability of nanobodies to accurately identify and quantify specific protein markers in complex biological matrices. This capability not only holds immense practical value for early disease diagnosis but also establishes a robust foundation for their broad application in disease marker detection. Furthermore, the potential uses of nanobody arrays may extend into therapeutic research and the multiplexed detection of diverse analytes. This broad scope of applications underscores the versatility of nanobody arrays. In conclusion, the precise immobilization of nanobodies, accomplished through cutting-edge enzymatic methods, further broadens the utility of nanobody arrays in life science research, marking a significant stride in both diagnostic and therapeutic domains. Overall, our findings demonstrate the potential of this novel nanobody immobilization method in creating highly sensitive, specific, and cost-effective antibody arrays. This approach holds significant promise for advancing methodologies in various biological and clinical applications.

## Data Availability

Data are contained within the article and Supplementary Materials.

## Supplementary Materials

The following supporting information can be downloaded at: https://www.mdpi.com/article/10.3390/molecules29020366/s1, Supplementary Note: Protein sequences; Figure S1: (a) SDS-PAGE: From left to right, the two bands represent the nanobody (15.3 kDa) and eGFP (32.5 kDa) respectively. (b) Native gel: From left to right, the bands represent eGFP and the eGFP-nanobody complex, stained with Coomassie Brilliant Blue; Figure S2: Schematic representation of the functionalization of glass substrates using click chemistry. (a) Azidation of the aminosilane surface with ImSO2N3·HCl; (b) Washing and drying; (c) Reaction of DBCO-PEGn-Mal with N3-modified glass slides; (d) Immobilization of bifunctional peptides with elastin-like peptides on the glass substrate, such as GL-ELP20-Cys; (e) Buffer washing and drying; (f) Nanobodies with NGL tags reacting with GL on the substrate surface and immobilization facilitated by *Oa*AEP1 catalysis; Figure S3: Fluorescence imaging of eGFP for specific nanobody recruitment and non-specific recruitment. (a) The green image of the cover slip area confirms that the observed fluorescence signal indeed originates from eGFP. Control experiments: (b,c) are with and without the addition of *Oa*AEP1 in the nanobody solution, respectively, dripped onto substrates not coated with GL-ELP20; Figure S4: Grayscale analysis of SDS-PAGE. (a) Grayscale profile analysis of each lane from top to bottom, as shown in the figure. The green solid line represents the original cell lysate, while the gray dashed line represents the lysate treated with the nanobody array. (b) Specific grayscale values of different lanes: Grayscale values of individual lanes arranged from top to bottom. The red box highlights the areas where a significant reduction in grayscale is observed after treatment with the nanobody array. The green solid line indicates the original cell lysate, and the gray dashed line indicates the lysate treated with the nanobody array; Table S1: Abbreviations.
