# Supplementary material for: Enzymatic Protein Immobilization for Nanobody Array
Source: Molecules. 2024 Jan 11;29(2):366. doi: 10.3390/molecules29020366 (PMC10820937; doi:10.3390/molecules29020366)
Supplement: Supplementary file 1 [file molecules-29-00366-s001.zip › molecules-2791840-supplementary.pdf]

Supplementary Information

Enzymatic Protein Immobilization for Nanobody Array

Zhuojian Lu, Rui Ge, Bin Zheng, and Peng Zheng*

State Key Laboratory of Coordination Chemistry, Chemistry and Biomedicine Innovation Center, School of Chemistry and Chemical Engineering, Nanjing University, Nanjing, Jiangsu, 210023, P. R. China

*Corresponding author: Correspondence and requests for materials should be addressed to P.Z. (Email: pengz@nju.edu.cn)

This Supplementary Information Includes:

Supplementary Note 4

Protein sequences..4

Supplementary Figures..5

Figure S1...5

Figure S2...6

Figure S3...6

Figure S4...7

Supplementary Note

Protein sequences

nanobody (7SAI)- $(ELP)_8$-NGL- His_8

QVQLVESGGGLVQAGGSLRLSAAASGRTFSTSAMGWFRQAPGREREFVAA

ITWTVGNTIYGDSMKGRFTISRDRTKNTVDLQMDSLKPEDTAVYYATARSR

GFVLSDLRSVDSFDYKGQGTQVTVSRSVPGVGVPGVGVPGEGVPGVGVPG

VGVPGVGVPGVGVPGEGVPGGLRSNGLHHHHHHHH

eGFP - His_8

MVSKGEELFTGVVPILVELDGDVNGHKFSVSGEGEGDATYGKLTLKFICTT

GKLPVPWPTLVTTLTYGVQCFSRYPDHMKQHDFFKSAMPEGYVQERTIFFK

DDGNYKTRAEVKFEGDTLVNRIELKGIDFKEDGNILGHKLEYNYNSHNVYI

MADKQKNGIKVNFKIRHNIEDGSVQLADHYQQNTPIGDGPVLLPDNHYL

STQSALSKDPNEKRDHMVLLEFVTAAGITLGMDELYKRSRSNGLHHHHH

HHH

Supplementary Figures

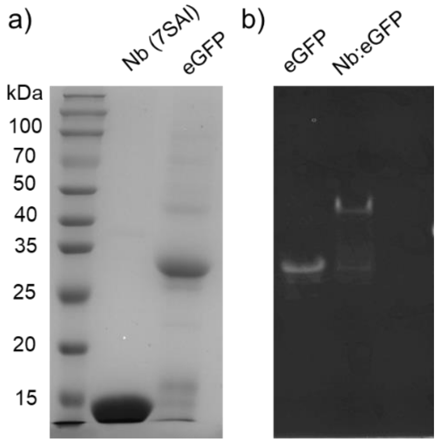

Figure S1. (a) SDS-PAGE: From left to right, the two bands represent the nanobody (15.3 kDa) and eGFP (32.5 kDa) respectively. (b) Native gel: From left to right, the bands represent eGFP and the eGFP-nanobody complex, stained with Coomassie Brilliant Blue.

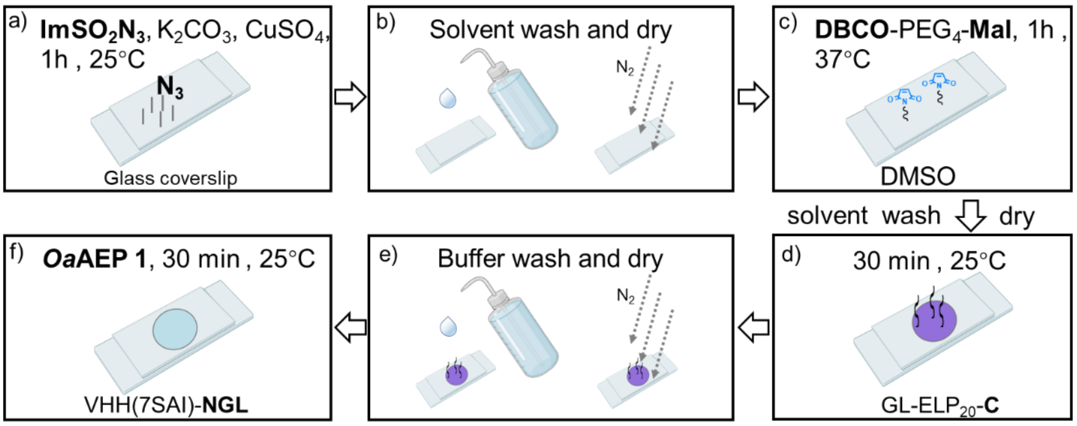

Figure S2. Schematic representation of the functionalization of glass substrates using click chemistry. a) Azidation of the aminosilane surface with $ImSO_2N_3{\cdot}HCl$; b) Washing and drying; c) Reaction of DBCO-PEG_n-Mal with N_3-modified glass slides; d) Immobilization of bifunctional peptides with elastin-like peptides on the glass substrate, such as GL-ELP_{20}-Cys; e) Buffer washing and drying; f) Nanobodies with NGL tags reacting with GL on the substrate surface and immobilization facilitated by *Oa*AEP1 catalysis.

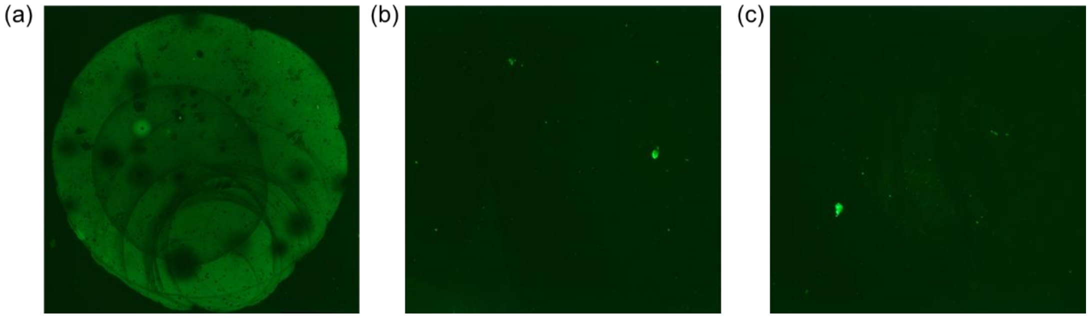

Figure S3. Fluorescence imaging of eGFP for specific nanobody recruitment and non-specific recruitment. a) The green image of the cover slip area confirms that the observed fluorescence signal indeed originates from eGFP. Control experiments: b) and c) are with and without the addition of *Oa*AEP1 in the nanobody solution, respectively, dripped onto substrates not coated with GL-ELP_{20}.

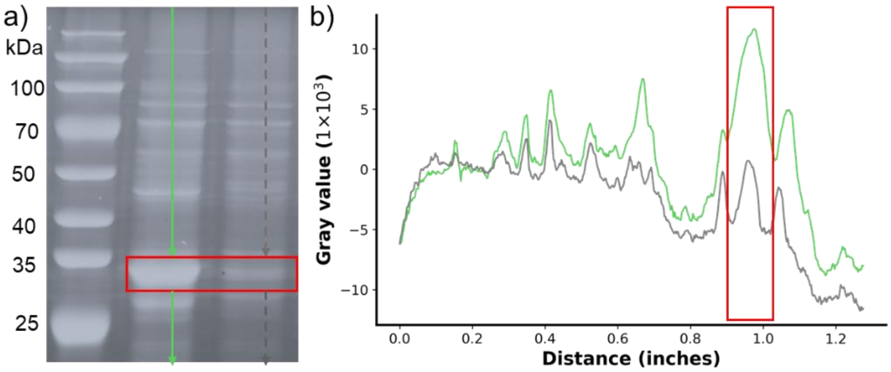

Figure S4. Grayscale analysis of SDS-PAGE. a) Grayscale profile analysis of each lane from top to bottom, as shown in the figure. The green solid line represents the original cell lysate, while the gray dashed line represents the lysate treated with the nanobody array. b) Specific grayscale values of different lanes: Grayscale values of individual lanes arranged from top to bottom. The red box highlights the areas where a significant reduction in grayscale is observed after treatment with the nanobody array. The green solid line indicates the original cell lysate, and the gray dashed line indicates the lysate treated with the nanobody array.

Table S1 Abbreviations

Abbreviation	Definition
Sortase A	SrtA
*Oa*AEP1	asparaginyl endopeptidases
LPXTG	Leu-Pro-X-Thr-Gly
GL	Gly-Leu
NGL	Asn-Gly-Leu
eGFP	enhanced green fluorescent protein
APTES	(3-Aminopropyl) triethoxysilane

DBCO	(dibenzocyclooctyne)-PEG_4-maleimide
DMSO	Dimethyl sulfoxide
LB	Luria-Bertani
ELP	elastin-like polypeptide(Val-Pro-Gly-X-Gly)
NA	numerical aperture
